# Identification of Genetic Elements Associated with *EPSPS* Gene Amplification

**DOI:** 10.1371/journal.pone.0065819

**Published:** 2013-06-10

**Authors:** Todd A. Gaines, Alice A. Wright, William T. Molin, Lothar Lorentz, Chance W. Riggins, Patrick J. Tranel, Roland Beffa, Philip Westra, Stephen B. Powles

**Affiliations:** 1 Australian Herbicide Resistance Initiative, School of Plant Biology, University of Western Australia, Crawley, Western Australia, Australia; 2 Weed Resistance Research, Bayer CropScience, Frankfurt am Main, Germany; 3 Crop Production Systems Research Unit, United States Department of Agriculture - Agricultural Research Service, Stoneville, Mississippi, United States of America; 4 Department of Crop Sciences, University of Illinois, Urbana, Illinois, United States of America; 5 Department of Bioagricultural Sciences and Pest Management, Colorado State University, Fort Collins, Colorado, United States of America; University of Arizona, United States of America

## Abstract

Weed populations can have high genetic plasticity and rapid responses to environmental selection pressures. For example, 100-fold amplification of the 5-enolpyruvylshikimate-3-phosphate synthase (*EPSPS*) gene evolved in the weed species *Amaranthus palmeri* to confer resistance to glyphosate, the world’s most important herbicide. However, the gene amplification mechanism is unknown. We sequenced the *EPSPS* gene and genomic regions flanking *EPSPS* loci in *A. palmeri*, and searched for mobile genetic elements or repetitive sequences. The *EPSPS* gene was 10,229 bp, containing 8 exons and 7 introns. The gene amplification likely proceeded through a DNA-mediated mechanism, as introns exist in the amplified gene copies and the entire amplified sequence is at least 30 kb in length. Our data support the presence of two *EPSPS* loci in susceptible (S) *A. palmeri*, and that only one of these was amplified in glyphosate-resistant (R) *A. palmeri*. The *EPSPS* gene amplification event likely occurred recently, as no sequence polymorphisms were found within introns of amplified *EPSPS* copies from R individuals. Sequences with homology to miniature inverted-repeat transposable elements (MITEs) were identified next to *EPSPS* gene copies only in R individuals. Additionally, a putative *Activator* (*Ac*) transposase and a repetitive sequence region were associated with amplified *EPSPS* genes. The mechanism controlling this DNA-mediated amplification remains unknown. Further investigation is necessary to determine if the gene amplification may have proceeded via DNA transposon-mediated replication, and/or unequal recombination between different genomic regions resulting in replication of the *EPSPS* gene.

## Introduction

Gene amplification, the reiteration of a coding segment resulting in one or more additional gene copies, is known to be a common process in the evolutionary history of plants and is vital for generating genomic diversity [1]. In addition to being a mechanism of adaptive evolution in mammalian cancer cells [2], bacteria [3], and arthropods [4], gene amplification is an important adaptive mechanism for antibiotic resistance, and the increased expression can offset fitness penalties associated with some resistance mechanisms [5]. Gene amplification and the resulting proportional increase in transcript levels has been implicated in insecticide resistance evolution in 10 different arthropod species, both for genes having a role in increased insecticide metabolism and for genes encoding proteins inhibited by insecticides (reviewed by [4]). Hence, numerous cases have been demonstrated where gene amplification has facilitated adaptive evolution.

Gene amplification is also an adaption in plants conferring resistance to the herbicide glyphosate [6]. Glyphosate is the world’s most important and widely used herbicide and persistent usage is resulting in resistance evolution [7]. An *Amaranthus palmeri* population highly resistant to glyphosate was found to have from 40- to 100-fold amplification of the 5-enolpyruvylshikimate-3-phosphate synthase (*EPSPS*) gene, and *EPSPS* gene hybridization signals were observed on each *A. palmeri* chromosome using fluorescence *in-situ* hybridisation [6]. The *EPSPS* gene produces EPSPS, essential in the synthesis of aromatic amino acids, and EPSPS is inhibited by glyphosate [8]. Increased *EPSPS* expression confers glyphosate resistance [9], and in *A. palmeri*, the extra EPSPS produced from the amplified gene copies are predicted to enable the plants to survive high glyphosate doses. The inheritance of this gene amplification is complex, as *EPSPS* copy number in progeny can vary substantially from parental copy number [10], and *EPSPS* gene amplification and glyphosate resistance can be transferred to related *Amaranthus* species through cross-pollination [11]. Amplification of the *EPSPS* gene has also recently been associated with glyphosate resistance in a *Lolium* population [12] and in *A. tuberculatus* populations [13].

The mechanistic processes involved in large-scale gene amplification conferring herbicide resistance are currently unknown. A proposed hypothesis for *EPSPS* gene amplification in *A. palmeri* is the activity of a mobile genetic element (MGE) [6]. Transposable elements (transposons) are one type of MGE and generate genetic diversity by moving within the genome [14], [15]. Transposons can be grouped into two classes, those that replicate through an RNA intermediate (class 1, retrotransposons) and those that replicate as DNA through a conservative cut-and-paste mechanism (class 2) [16]. Class 2 transposons can increase in copy number and contribute to genome expansion via two mechanisms, 1) transposing from one of two recently replicated chromatids into an un-replicated target site [17], and 2) through gene conversion, a gap repair mechanism that restores a copy of the original sequence to the empty donor site [18], [19]. Class 2 elements can be autonomous, encoding a transposase necessary for replication, or non-autonomous, generally derived from an autonomous element through deletion of internal sequences [16]. Together, transposable elements comprise a large part of the genome of higher organisms and have had major and recent effects on plant genome evolution and organization [16], [20].

Gene amplification may also be due to incorrect recombination or double-strand DNA break repair with subsequent tandem duplications as observed in bacteria, yeast, cancer cells, and plant cell cultures [21], [22], [23], [24]. However, the basis of the amplification mechanism in glyphosate-resistant *A. palmeri*, how rapidly the initial amplification occurred, and why inheritance of the elevated copy number is difficult to predict [10] are all unanswered questions. If gene amplification occurred via transposon activity, genomic regions flanking amplified *EPSPS* genes may provide evidence of transposon insertions and the presence of introns may provide evidence for the class of transposon responsible. Therefore, experiments were conducted in *A. palmeri* to 1) sequence amplified *EPSPS* genes and genomic regions flanking *EPSPS* loci using two high-throughput sequencing platforms (454 pyrosequencing and Illumina), 2) identify transposons, other repetitive sequences, and intron sequence diversity, and 3) search for evidence of tandem gene duplications.

## Results

### EPSPS Intron Analysis

#### Sequencing Introns

**Figure 1:**
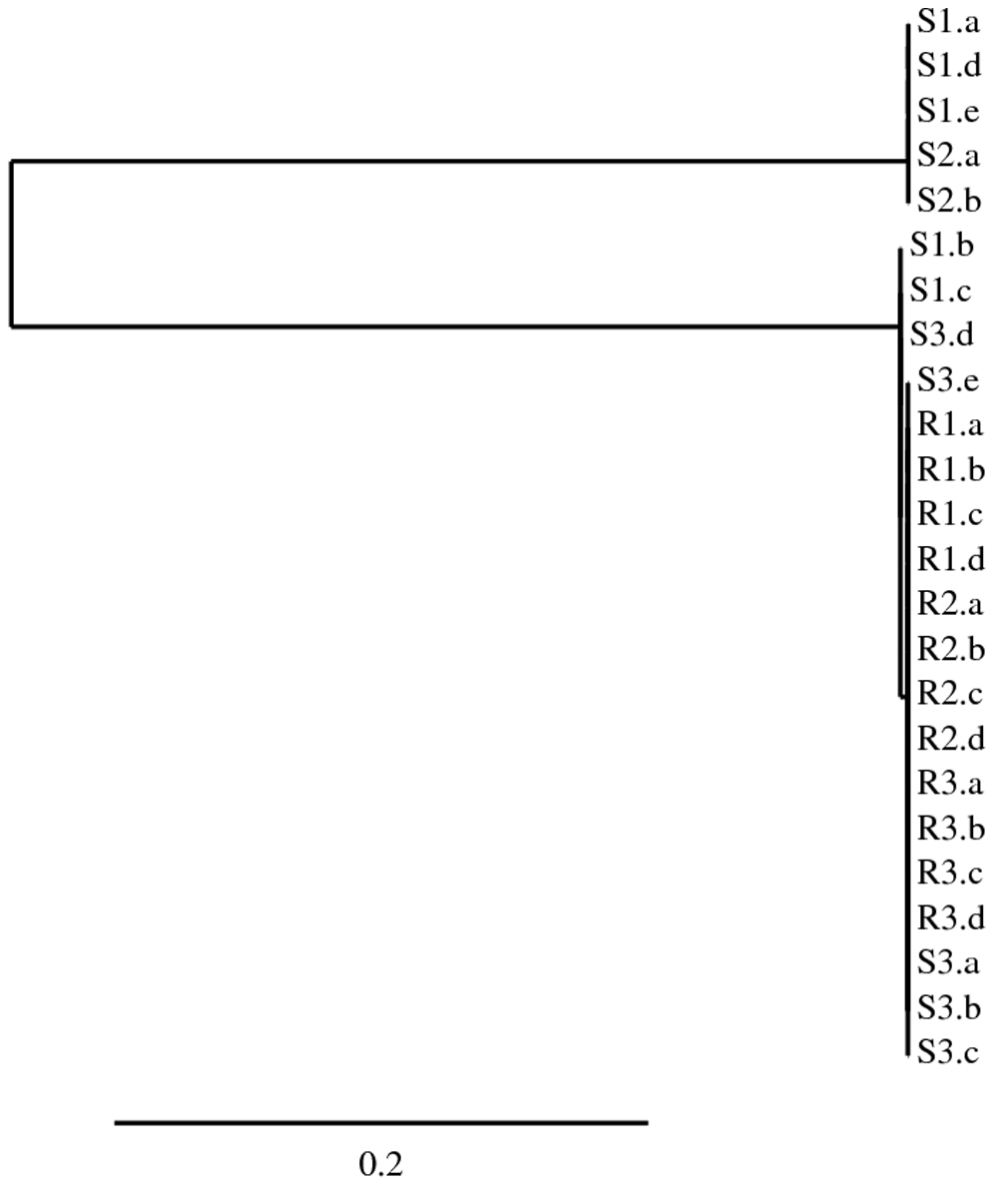
Phylogenetic tree of cloned and sequenced PCR products (individual clones labeled a-e) spanning two introns of the *EPSPS* gene in three glyphosate-resistant (R) and three susceptible (S) *A. palmeri* individuals supports the existence of two *EPSPS* loci in *A. palmeri*, and the amplification of one *EPSPS* locus in R genomes. Scale bar represents the number of nucleotide substitutions per site.

We first examined whether intron sequences were present in *EPSPS* gene sequences from the previously reported [6] glyphosate-resistant *A. palmeri* population from Georgia, USA (GA-R) and a glyphosate-susceptible population (GA-S). PCR amplification using primers (Table S1) spanning two predicted introns produced 765 to 767 bp amplicons from GA-R and GA-S (Figure S1), longer than the mature mRNA sequence length of 331 bp. These amplicons were cloned and sequenced and intron sequences were found in both GA-R and GA-S (Figure S1). Intron boundary splice sites matched the intron boundaries in the *Arabidopsis EPSPS* gene [25]. A phylogenetic tree shows that the GA-R sequences cluster with some GA-S sequences, and other GA-S sequences form a second group (Figure 1). All GA-R sequences contained an *Xho* I restriction site in intron 5, while GA-S sequences were polymorphic for this restriction site (Figure S1). Intriguingly, the intron sequences from all GA-R clones were identical, while polymorphisms including an A deletion and an AAC insertion were found in GA-S clones (Figure 1, S1). The S population clearly formed two groups of *EPSPS* sequences (Figure 1), consistent with previously reported evidence for two *EPSPS* loci in S *A. palmeri* [26]. Our data suggest that resistance to glyphosate resulted from amplification of only one allele from the two *EPSPS* loci. It is not known whether expression differs between these two putative *EPSPS* loci or whether there are any enzymatic differences in the gene products.

#### Quantitative PCR

Primers for qPCR were designed based on the intron sequences (Table S1). Because individuals from this GA-R *A. palmeri* population are known to have *EPSPS* copy numbers of 100-fold or more relative to GA-S, qPCR specific to the intron sequence was used to estimate whether any loci could be detected in GA-R individuals that did not carry introns. Intron-specific primers produced the same estimate of *EPSPS* genomic copy number as exon-specific primers for all individuals tested (Table 1). These data indicate that all or nearly all of the amplified *EPSPS* copies in GA-R contain introns.

**Table 1:** *EPSPS* genomic copy number measured using qPCR primers within an intron is similar to copy number measured using qPCR primers within an exon for R and S individuals.

	-*EPSPS*:*ALS* Relative Genomic Copy Number-					
	R1	R2	R3	S1	S2	S3
Exon	108 (4.4)	84 (8.6)	60 (0.5)	1.0 (0.06)	0.9 (0.02)	0.9 (0.01)
Intron	106 (1.2)	73 (3.0)	57 (5.1)	0.8 (0.04)	0.9 (0.07)	1.0 (0.07)

Data are means with standard errors in parentheses.

### Genomic Sequencing

#### 454 Pyrosequencing

Genomic DNA from a highly glyphosate-resistant GA-R individual with 86-fold *EPSPS* relative gene amplification (relative copy number determined by qPCR [6]) was *de novo* sequenced using the Roche GS-FLX 454 platform. More than 800,000 reads with an average length of 560 bp were obtained from shotgun genomic sequencing and assembled into contigs. As expected, numerous hits to the *EPSPS* gene were obtained. Amplification was specific to the *EPSPS* gene relative to other herbicide target-site genes (Table 2), and the ratio of *EPSPS* reads to acetolactate synthase (*ALS*) reads obtained from 454 sequencing (150-fold more) was similar to the *EPSPS*:*ALS* ratio from qPCR (86-fold). A total of 3,278 individual sequence reads were assembled into one large contig of 14,268 bp encompassing the full-length genomic *EPSPS* gene. Numerous transposable elements were identified in the genomic sequence, the major proportion of which was long terminal repeat (LTR) retrotransposons. Several other general categories of transposable elements were also identified, similar to that reported by Lee *et al*. [27] for *A. tuberculatus*. Sequences were identified flanking *EPSPS* on the 5′ and 3′ ends (Table 3) with high similarity to miniature inverted-repeat transposable elements (MITEs) characterized in the *Oryza* Repeat Database [28]. A 13 bp imperfect Terminal Inverted Repeat (TIR) and a 3 bp (TAA) duplication were identified in the 454 sequence immediately adjacent to the MITE-homologous regions on the 5′ and 3′ ends of the *EPSPS* gene (Figure S2A).

**Table 2:** Hits to *EPSPS* and other genes encoding herbicide target sites from 454 genomic sequencing in *A. palmeri*. BLAST searches performed against all raw reads and unigene only (contigs+singletons) databases.

Herbicide target	Hits to all raw reads	Hits to unigenes
Acetolactate synthase (*ALS*)	2	1 (32%)
5-enolpyruvylshikimate-3-phosphate synthase (*EPSPS*)	321	1 (100%)
4-hydroxyphenylpyruvate dioxygenase (*HPPD*)	3	4 (80%)
Protoporphyrinogen oxidase 2 (*PPX2*)	1	2 (24%)
Protoporphyrinogen oxidase 1 (*PPX1*)	2	2 (29%)

Raw read hits were normalized to 1000 bp for each gene query. Unigene searches include full length coding sequences for each gene and percent coverage in parentheses. All BLAST searches were conducted with an E-value cutoff of 10^−50^ and >90% sequence similarity. Sources and sizes of queries:

*ALS*: complete cds from *A. tuberculatus* (Genbank EF157819; 2010 bp).

*EPSPS*: complete cds from *A. palmeri* (Genbank FJ861243; 1557 bp).

*HPPD*: complete cds from *A. tuberculatus* (Genbank JX259255; 1471 bp).

*PPX2*: complete cds from *A. tuberculatus* (Genbank EU024569; 1608 bp).

*PPX1*: complete cds from *A. tuberculatus* (Genbank DQ386115; 1659 bp).

**Table 3:** Sequences in genomic regions flanking *EPSPS* in *A. palmeri* have similarity to known transposable elements in rice [28].

Sequence	*Oryza* Repeat Database Hit	Strand	Length of Similarity (bp)	Hit Score	% Match
5′ MITE	ORSgTEMT00500849, putative MITE, Gaijin/Gaigin-like	+	155	122	60
	ORSgTEMT01602459, putative MITE, MITE-adh, type B-like	+	160	128	57
3′ MITE	ORSgTEMT01600913, putative MITE, MITE-adh, type B-like	–	209	170	58
	ORSgTEMT02900113, putative MITE, MITE-adh-4-like	–	298	186	57
	ORSgTEMT00100233, putative MITE, Tourist-like	–	140	169	62

Sequences were identified bordering *EPSPS* in a contig assembled from 454 sequencing of GA-R gDNA.

#### Fosmid library sequencing

**Figure 2:**
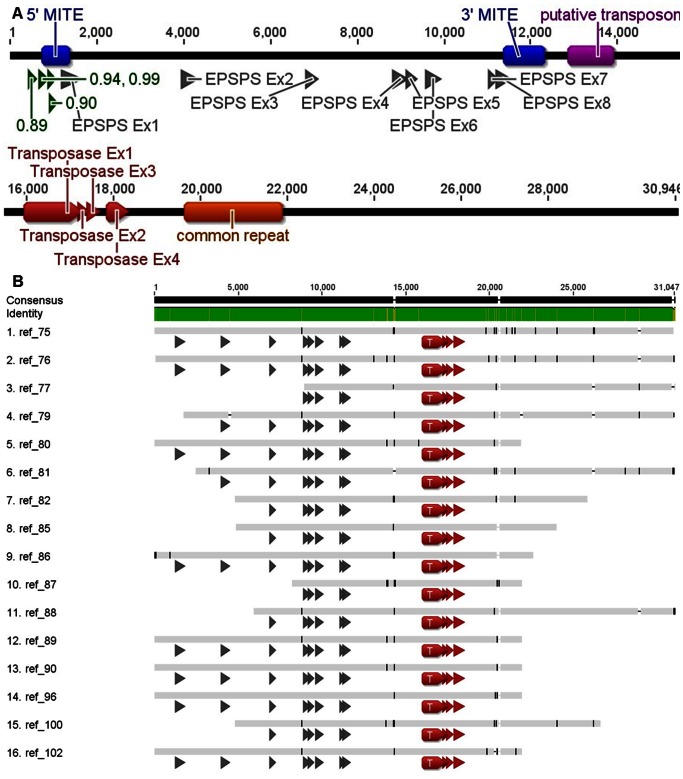
Consensus sequence of *A. palmeri EPSPS* from 16 MS-R fosmids. A) exons for *EPSPS* (gray) and a putative *Ac* transposase (red), putative promoters (green; numbers indicate promoter prediction score, 1 is maximum possible), MITE-homologous sequence (blue), a putative transposon (purple), and a repetitive sequence motif (orange). B) Alignment of the consensus *A. palmeri EPSPS* and flanking genomic region sequences for 16 fosmids from MS-R. *EPSPS* exons (gray arrows) from 1 to 12,000 bp, and exons of a putative *Ac* transposase (red) from 16,000 to 19,000 bp. Green color in identity bar indicates identical sequences in all fosmids and brown indicates polymorphisms.

A fosmid library was constructed from genomic DNA of an *A. palmeri* individual with 80-fold increased *EPSPS* expression (as determined by qPCR). To compare geographically distant populations and different sequencing techniques, this individual was isolated from a second glyphosate-resistant population found in Mississippi, USA (MS-R). Sixteen MS-R fosmid clones containing *EPSPS* sequence were identified and sequenced with Illumina 50 bp single reads. Sequence coverage was insufficient to permit initial individual assembly, so all sequence reads were first pooled to create a reference sequence (Figure 2A). Next, barcoded sequence reads for each fosmid were assembled individually. Alignment of the contigs from individual assemblies to the reference sequence revealed very few sequence differences among fosmids (Figure 2B). End points of the fosmids were determined by lack of assembly to the consensus beyond a certain point, inclusion of vector sequence with contigs containing insert, and confirmation by PCR. Fosmid insert sequence coverage ranged from 59.8% to 99.8%, aligned reads per fosmid insert ranged from 813 to 46,413, and estimated depth ranged from 1.3-fold to 104-fold coverage.

Alignment of these 16 clones produced a consensus sequence (GenBank Accession JX564536) of 30,945 base pairs containing the entire *EPSPS* sequence including 8 exons and 7 introns (Figure 2A). Substantially more sequence was obtained downstream (19,464 bp) than upstream (1,252 bp) of the gene. The *EPSPS* gene was 10,229 bp long, with the expected coding sequence length of 1,557 bp (*A. palmeri EPSPS* GenBank Accessions FJ861242.1 and FJ861243.1) containing 8 exons of 333, 245, 154, 215, 118, 211, 62, and 219 bp, and 7 introns of 2416, 2624, 1856, 78, 356, 1242, and 100 bp. All exons were the same sizes as those from both the petunia (*Petunia hybrida*) and *Arabidopsis EPSPS* genes, with the exception of the first exon containing the chloroplast transit peptide (327 bp in petunia and 339 bp in *Arabidopsis*) [25], [29]. The *A. palmeri* coding region of 10.2 kb is longer than petunia (7.4 kb) and *Arabidopsis* (2.5 kb) due to longer intron length; for example, the first intron is 1.3 kb in petunia and only 87 bp in *Arabidopsis* [25], [29].

Predicted promoter motifs and the previously identified MITE-homologous sequences were identified (Figure 2A). A putative transposase, revealed by BLASTn and BLASTp analysis to be similar to several *Activator* (*Ac*) transposases, was identified 4.5 kb downstream of *EPSPS* exon 8 (Figure 2A). It is not known if this putative *Ac* transposase is expressed or produces a functional gene product. A 256 bp imperfect inverted repeat, referred to as a putative transposon, was identified 1,432 bp downstream (Figure 2A, S3A, S3B) from *EPSPS*. Assembly of the sequence reads for each individual fosmid did not reveal a sequence divergence point, as all fosmids obtained had nearly identical overlapping sequences (Figure 2B). No tandem duplicated *EPSPS* genes were observed in the 16 sequenced fosmid inserts. Additional Sanger sequencing for two fosmids, AW88 and AW96, verified the accuracy of the sequence data obtained by the Illumina sequencing procedure (data not shown).

**Figure 3:**
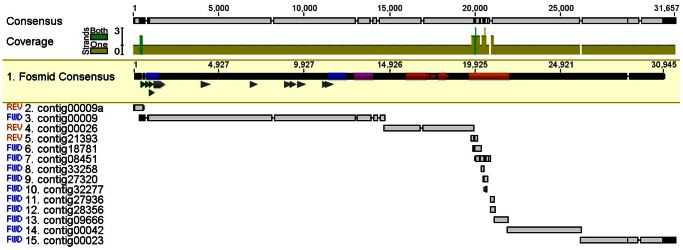
Assembly of 454 contigs to the fosmid consensus sequence. Contigs were identified by running a BLAST of the 454 data against the fosmid consensus sequence. Top sequence highlighted in yellow is the fosmid consensus sequence, remaining sequences are from the 454 data, and sequence at the top is the consensus of the fosmid and 454 data. The *EPSPS* exons (gray), MITE-homologous sequences (blue), a putative transposon (purple), a putative *Ac* transposase (red), putative promoters (green triangles), and a repetitive sequence motif (orange) are indicated.

Aligning 454 contigs to the fosmid consensus sequence revealed very high sequence similarity (Figure 3). Within the entire *EPSPS* gene, the 454 consensus and fosmid consensus differed by only 1 nucleotide, a single T insertion within intron 3 found in the fosmid consensus (Figure 3). Additional contigs from the 454 assembly aligned to the downstream fosmid reference (Figure 3), and these contigs also had a high number of hits (Table S2), confirming that these sequences were amplified in addition to the *EPSPS* gene sequence in both populations. Some contigs had even more hits than the *EPSPS* contig (Table S2), suggesting that these sequences occur elsewhere in the genome in addition to flanking amplified *EPSPS* genes. The fosmid consensus sequence from exon 4 to exon 6, crossing two introns, was identical to the sequence obtained both by 454 sequencing and by PCR from the GA-R population.

One notable feature was a repetitive sequence motif (identified as ‘common repeat’ in Figure 2A). The motif was identified by stacking of a large number of Illumina sequence reads over a 2.1 kb region after assembly of Illumina reads to the reference sequence (Figure S4). Several 454 contigs aligned to the motif (Figure 3) and had a higher number of hits than the *EPSPS* contig (Table S2). Observed restriction fragment lengths of fosmid inserts did not match predicted lengths based on the sequence within the assembled 2.1 kb ‘common repeat’ region. A repeated motif of 551 bp occurred twice within the ‘common repeat’ region, and it contained an internal 26 bp direct repeat and an internal 15 bp inverted repeat. The actual length of this section is likely longer than assembled in the reference, and additional sequencing will be necessary to resolve this region.

The 454 *EPSPS* sequence (contig 00009) had nearly 100% sequence homology upstream of *EPSPS* with the fosmid library sequence until a divergence point just upstream of the 5′ MITE-homologous sequence (Figure 3, S2A). The 5′ TIR and TAA duplication identified in the 454 sequence (Figure S2A) were not present in the fosmid sequence. The fosmid sequence contains a TATA box (Figure S2A) that is absent from the 454 sequence. Additionally, the assembled sequence in the 454 contig 00009 upstream of the TIR aligned in reverse orientation to the assembled fosmid sequence in this position (contig 00009a, Figure 3). The 454 sequence assembly was confirmed by PCR on GA-R individuals, as primers specific to the 454 contig 00009 sequence (Table S1) amplified products of the predicted size with a reverse primer in *EPSPS* exon 1, while primers specific to the fosmid (MS-R) sequence produced no PCR products with a reverse primer in *EPSPS* exon 1 (Figure S2B). The 454 sequence and fosmid library sequence had 100% sequence homology around the 3′ MITE-homologous sequence, with no divergence points identified (Figure 3, S2A).

### Gene Amplification Structure Analysis

**Figure 4:**
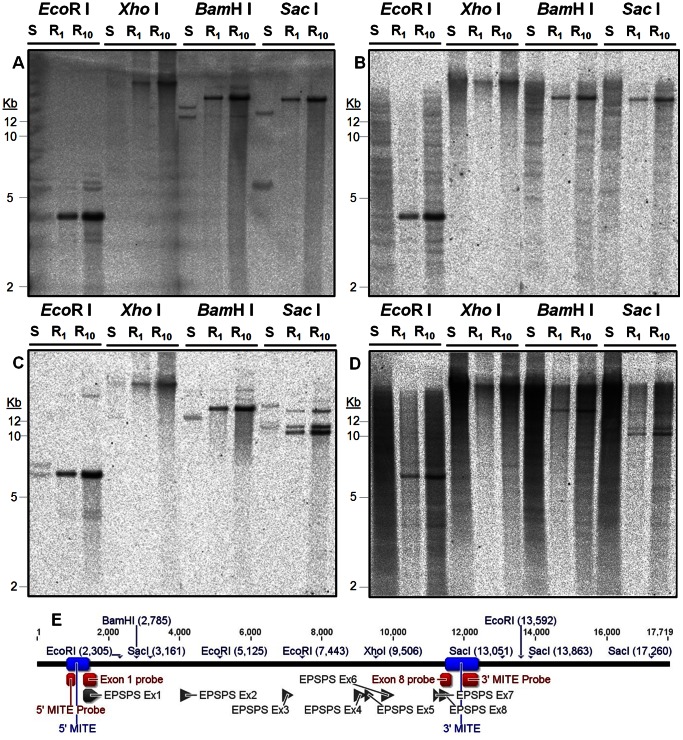
Southern blot analysis of *EPSPS* gene amplification in GA-S (S, 20 µg gDNA) and GA-R (R1, 1 µg gDNA and R10, 10 µg gDNA) *A. palmeri*. Hybridizations with ^33^P-dCTP labeled probes for A) *EPSPS* first exon, B) 5′ MITE, C) *EPSPS* last exon, and D) 3′ MITE; E) probe and exon locations and expected restriction sites.

DNA blot hybridizations were conducted using probes in Exon 1 and Exon 8 of *EPSPS* (the first and last exons, respectively) on GA-R and GA-S restriction-digested DNA. The expected pattern of much higher hybridization signal intensity in GA-R than in GA-S was observed (Figure 4A, C). Patterns in GA-S support the existence of 2 distinct *EPSPS* loci, consistent with the intron sequence phylogeny results (Figure 1). If *EPSPS* loci were arranged as tandem duplications in GA-R with inter-genic regions ≤20 kb, then we had expected to observe both the Exon 1 and the Exon 8 probes hybridizing to the same fragment, with the assumption that the expected flanking restriction sites (Figure 4E) would have been lost in a tandem duplication event. This was not observed, however, as no bands were common between Exon 1 and Exon 8 for all four restriction enzymes in GA-R. Combining the sizes of the observed *Bam*H I bands suggests the size of the amplified *EPSPS* locus is at least 30 kb (Figure 4A, C).

Additional hybridizations (Figure 4B, D) were conducted with probes for the MITE-homologous sequences identified next to the *EPSPS* gene in the 454 and fosmid library sequencing (probe sequence shown in Figure S2A). Hybridization with these probes occurred to the same size fragments as the respective Exon 1 and Exon 8 probes in GA-R, but the MITE-homologous probes did not hybridize to the same fragments as Exon 1 and Exon 8 in GA-S (Figure 4B, D). Additionally, the MITE-homologous probes hybridized to multiple fragments of the same size in both GA-S and GA-R, with additional fragments observed in GA-S but not in GA-R (Figure 4B, D). PCR experiments indicated both 5′ and 3′ MITE probes could be amplified from GA-S and GA-R (Figure S5A, B). Amplification from both 5′ and 3′ MITE-homologous sequences to *EPSPS* occurred only in GA-R (Figure S5C, D) and not in 16 GA-S individuals (Figure S5E, F, G) or in 5 S individuals from a North Carolina *A. palmeri* population (Figure S5H). Therefore, it appears that the genomic sequence flanking *EPSPS* within approximately 500 bp in GA-R is different from the two loci predicted in GA-S due to the insertion of sequence that also occurs at other locations in the genome.

Additional bands with lower intensity were observed using Exon 1 and 8 probes in *Eco*R I and *Bam*H I digested gDNA, both shorter and longer than expected (Figure 4A, C, E). This could indicate the existence of a few amplified loci with length polymorphisms due to other insertions or deletions. All other observed hybridizations were consistent with expected results based on the predicted restriction sites obtained from fosmid library sequencing (Figure 4E), except for the results obtained with the *Sac* I digest and the Exon 8 probe in GA-R. Three *Sac* I restriction sites were predicted downstream from Exon 8 prior to the first expected *Bam*H I site (Figure 4C, E). However, three major bands (10, 11, and 14 kb) were observed for *Sac* I and a single major band was observed for *Bam*H I (15 kb) (Figure 4C). Both *Sac* I sites are located within a large inverted repeat (Figure S3A) referred to as a putative transposon (Figure 2A) and if none, one, or two *Sac* I sites were disrupted, the expected fragment sizes using the Exon 8 probe would be 9.9, 10.7, and 14 kb, matching the observed sizes (Figure 4C). As *Sac* I is sensitive to cytosine methylation at GAGmCTC, the observed restriction fragment length polymorphism could be due to differences in DNA methylation status, or it could suggest that some sequence differentiation has occurred among amplified loci within the putative transposon.

## Discussion

DNA-mediated amplification of the *EPSPS* gene has occurred recently in glyphosate-resistant *A. palmeri*. No intron sequence variation was detected within GA-R and MS-R individuals, two *EPSPS* loci were detected in the S *A. palmeri* genome, and it appears that only one *EPSPS* locus was amplified in R individuals. Amplification of the entire *EPSPS* gene is supported by fosmid library data, as all fosmids positive for *EPSPS* contained introns. Both Southern blot and fosmid sequencing results suggest that no small tandem *EPSPS* duplications are present, although tandem duplications greater than 30 kb could be possible. Very few polymorphisms were detected among fosmids sequenced using Illumina short read technology. The fosmid, 454, and Southern blot data support DNA-mediated amplification in that at least 30 kb of sequence containing the *EPSPS* gene has been amplified. An RNA-mediated gene amplification process would likely insert a mature mRNA, i.e., with introns spliced out, into the genomic DNA. A process inserting an immature *EPSPS* mRNA, with introns still intact, would presumably leave short 5′ and 3′ untranslated regions, and neither RNA-mediated scenario is consistent with the fosmid library sequencing results. The haploid genome size of *A. palmeri* is estimated to be approximately 450 Mbp [30]. Therefore a 100-fold amplification of a 30 kb fragment would represent 3 million additional bp, a 0.67% increase in total genome size.

Sequence differences were observed in the first 500 bp flanking both sides of *EPSPS* between GA-S and both GA-R and MS-R individuals, where sequences with homology to known MITEs were associated with the *EPSPS* gene only in glyphosate-resistant individuals. The MITE-homologous sequences were detected elsewhere in the S and R genomes, but not next to the *EPSPS* gene in S. MITEs are one type of non-autonomous class 2 (DNA-mediated) transposons, characterized by TIR of between 10 and 20 bp and Target Site Duplications (TSD) generally of 3 bp (including TAA), and often inserting in AT rich regions [31], [32]. Although MITEs have not previously been shown to acquire and duplicate functional gene sequences, *Mutator*-like elements (MULEs) in *Arabidopsis* [33] and rice [34] commonly acquire and duplicate short host gene fragments. Both TIR and a TSD-like sequence motif were identified adjacent to the MITE-homologous sequences flanking amplified *EPSPS* genes in the GA-R population, and different sequences were found upstream from the 5′ MITE-homologous sequence between the GA-R and MS-R populations. The absence in the MS-R fosmid sequence of the TIR and TSD identified in the GA-R 454 sequence, and the reversed alignment of GA-R 454 sequence to the MS-R fosmid sequence upstream of the TIR, may be due to population differences between GA-R and MS-R, may indicate separate origins of the gene amplification mechanism, or may indicate the border of the amplified unit. Given that the length of the amplification extends at least 18 kb past the 3′ MITE-homologous sequence, we cannot conclude that the identified MITE-homologous sequences are mechanistically responsible for the gene amplification and the amplification mechanism remains unknown. Further investigation is necessary to determine if the MITE-homologous sequences are simply passengers in the amplified DNA sequence, or if they have a role in the amplification mechanism.

The active MITE family known as *mPing* was first identified in rice [35] and can rapidly increase in copy number each generation without negative effects. The element preferentially inserts close to genes (within 5 kb) but less frequently in exons or introns [36]. Novel *mPing* insertions in 5′ and 3′ flanking regions, often within 1 kb, can influence gene expression regulation, particularly resulting in increased gene expression under stress conditions [37]. Increased *mPing* activity was associated with adaptation to an extremely different temperate environment during rice domestication [35], suggesting that MITE amplification can generate adaptive genetic diversity. A MITE in *Brassica* was found to preferentially accumulate in gene regulatory regions but not in coding regions [38], which is also the case in the present study. MITEs were found to flank 58% of genes in the rice genome [39], and 15% of MITEs have presence/absence polymorphisms among selected rice cultivars.

Both transposons and repetitive sequences have been found flanking amplified genes conferring insecticide resistance. An insecticide resistant *Culex* mosquito population had duplicated copies of a cytochrome P450 (CytP450) gene, over 260-fold increased expression, and the insertion of a MITE-like element was found upstream of both copies [40]. Daborn *et al*. [41] showed that increased expression of a CytP450 gene in *Drosophila* was sufficient for resistance, and the increased expression was due to insertion of an *Accord* transposable element upstream of the gene. Both an increase in copy number and insertions of transposable elements in regulatory regions have contributed to insecticide resistance, occurring in multiple steps and permitting adaptation of *D. melanogaster* to insecticides [42]. Currently it is unknown whether the MITE insertion in the *Culex* population is conferring *cis*-mediated increased expression as in *Drosophilia*, but it could be possible, both for *Culex* and for *A. palmeri*. An esterase B1 gene was found to be amplified around 250-fold in *Culex* mosquitos [43]. The amplified gene had neighboring repetitive sequences that were also found in other parts of the R genome, and also found in the S genome, but not near the esterase gene. The esterase B1 genes were present as single copies in a 25 kb sequence that was highly conserved in amplified copies, and flanked by larger, more heterogenous regions, with the entire amplicon up to 100 kb in total. *Myzus* aphids had amplified esterase genes occurring on multiple chromosomes [44], and the authors postulated that this was due to reciprocal interchange between chromosomes, or possibly due to the activity of transposable elements.

In summary, amplification of the *EPSPS* gene in glyphosate-resistant *A. palmeri* has occurred through a DNA-mediated mechanism. Our data support the presence of two *EPSPS* loci in S *A. palmeri*, and only one *EPSPS* locus has been amplified in glyphosate-resistant *A. palmeri*. We have shown that sequences with homology to MITEs (non-autonomous class 2 transposons) are present in the genomes of both S and R individuals, but are associated with *EPSPS* gene copies only in R individuals from two different populations. Additionally, a predicted *Ac* transposase and a large repetitive sequence were found downstream of amplified *EPSPS* copies. The mechanism directing the DNA-mediated gene amplification remains unknown. The large size of the amplified sequence (>30 kb), association with various types of genetic elements, and the previously reported unpredictable copy number inheritance patterns [10] are all intriguing, and raise questions such as whether the *EPSPS* gene amplification is an inducible adaptive mutation via a transposon-mediated process. Additional bordering DNA sequence of amplified regions from *A. palmeri* and other plant species with *EPSPS* gene amplification [12], [13] should provide insight into candidate mechanisms such as DNA-mediated transposon activity and/or unequal recombination between different genomic regions resulting in replication of the *EPSPS* gene.

## Materials and Methods

### EPSPS Intron Analysis

#### Sequencing Introns

Genomic DNA (gDNA) was extracted from 3 glyphosate-resistant (GA-R) and 3 -susceptible (GA-S) *A. palmeri* individuals from Georgia, USA, as previously described [6]. Primers Ex4F and Ex6R1 (Table S1) were designed based on the *A. palmeri EPSPS* sequence (GenBank Accessions FJ861242.1 and FJ861243.1) to amplify from exon 4 to exon 6 of *EPSPS*, crossing 2 introns. The expected coding sequence from the cDNA was 331 bp long, and it was expected that longer amplicons would contain introns. PCR was conducted using Phusion High-Fidelity DNA Polymerase (New England Biolabs) in 25 µL reactions with 1X HF buffer, 200 µM each dNTP, 0.5 µM each primer, 0.5 Units polymerase, and 10 ng template DNA, with initial denaturation at 98°C for 30 sec and then 25 cycles with 98°C for 15 sec, 55°C for 15 sec, and 72°C for 15 sec. Amplicons were separated on 1% agarose gel and gel extracted for cloning. Amplicons were cloned using the StrataClone Blunt PCR cloning kit (Agilent) according to the manufacturer’s instructions. Plasmids were isolated (Qiagen Plasmid Mini Kit) from white clones, the presence of an insert was confirmed by *Eco*R I digestion, and positive plasmids were submitted for sequencing at the Australian Genome Research Facility (AGRF). Sequences were aligned and a phylogenetic tree was constructed using Phylogeny.fr [45].

#### Quantitative PCR

Primers In5F and Ex6R2 (Table S1) were designed to produce a 155 bp amplicon within the identified intron sequences for quantitative PCR (qPCR). Previously described primers and qPCR protocols [6] were used to measure *EPSPS* genomic copy number relative to the *ALS* gene. Primers within an *EPSPS* exon were used, which have previously shown in glyphosate-susceptible individuals an *EPSPS* copy number relative to *ALS* of one [6]. After verifying for amplification of the expected intron PCR product, the intron primers were used in addition to the exon primers to measure *EPSPS* genomic copy relative to *ALS* genomic copy number.

### Genomic Sequencing

#### 454 Pryosequencing

Genomic DNA was extracted from one individual from the GA-R population using the Plant DNEasy kit (Qiagen) for use in 454 pyrosequencing (schematic in Figure S6). The individual plant was selected because of its high *EPSPS* copy number (86-fold, relative to *ALS*, based on qPCR). One-half of a pico-titer plate was sequenced on the Roche GS-FLX 454 at the W.M. Keck Center for Comparative and Functional Genomics at the University of Illinois. Initial quality control was performed before base calling, and assembly was performed as previously described [27]. Hits to the *EPSPS* gene and to other herbicide target genes including *ALS*, 4-hydroxyphenylpyruvate dioxygenase (*HPPD*), and protoporphyrinogen oxidase (*PPX1* and *PPX2*) were compared to assess whether gene amplification was specific to *EPSPS*. Additionally, assembled contigs were searched for transposable elements using RepeatMasker (http://www.repeatmasker.org). All contig sequences were searched against the *Oryza* and *Arabidopsis* repeat libraries using default settings. Outputs were compared to results from similar searches performed with *A. tuberculatus* 454-derived sequence data [27].

### Fosmid Library and Sequencing

A fosmid library was prepared from gDNA from a different glyphosate-resistant *A. palmeri* population from Mississippi, USA (MS-R). A glyphosate-resistant individual was identified with 80-fold increased *EPSPS* expression as determined by qPCR [6]. Genomic DNA was extracted using the Masterpure DNA purification kit (Epicentre) and the fosmid library was constructed as described in Methods S1. Sanger sequencing of fosmid inserts was performed by the USDA-ARS GBRU. Fosmid insert DNA for Illumina library preparation was fragmented to 100–300 bp following the protocol for the dsDNA fragmentase (New England Biolabs), and fragmented DNA was prepared for sequencing using the NEXTflex DNA Sequencing kit (Bioo Scientific). Each fosmid was labeled with a barcode prior to pooling of the libraries for sequencing. Libraries were sequenced on an Illumina HiSeq 2000 with 50 bp single reads and the raw data were analyzed by the USDA-ARS GBRU. Assemblies were performed using Geneious Pro [46] and CLC Bio [47]. Promoters were identified using a neural network promoter predictor from the Berkley Drosophila Genome Project [48], using default parameters.

### Gene Amplification Structure Analysis

Genomic DNA from one GA-R and one GA-S individual was extracted and digested with restriction enzymes, transferred to a membrane, and hybridized with probes as described in Methods S1. Probes were hybridized in the order of *EPSPS* Exon 1, Exon 8, 5′ MITE, and 3′ MITE, and the blot was stripped after each hybridization (Methods S1).

## Supporting Information

Figure S1 Sequence alignment of *EPSPS* genomic sequence (exon 4 to exon 6) from representative glyphosate-resistant (R) and –susceptible (S) *A. palmeri* cloned PCR products. Primers Ex4F and Ex6R1 are underlined, and *Xho*I polymorphism is highlighted with a square. Intron sequences are in lower case.
(DOCX)

Figure S2 Alignment of fosmid consensus sequence (MS-R), 454 consensus sequence (GA-R), and the probes used for Southern blots (5′ MITE and 3′ MITE from GA-R). A 13 bp imperfect Terminal Inverted Repeat (TIR) is underlined with one non-matching base in lower case, and a 3 bp duplicated sequence similar to known Target Site Duplications (TSD) is shown in bold and italics.
(DOCX)

Figure S3 A 256 bp imperfect inverted repeat identified in fosmid sequence from MS-R gDNA; A, alignment showing identity between inverted repeat on ends of the putative transposon (Figure 4) and B, sequence of the inverted repeat.
(DOCX)

Figure S4 Assembly of Illumina reads to the fosmid reference sequence, and the presence of read stacking in a region from approximately 20,000 bp until 22,000 bp. The sequence in this region contains a repeated 551 bp sequence.
(TIF)

Figure S5 PCR evidence for existence of putative MITE sequences in both GA-R and GA-S, but present flanking *EPSPS* only in GA-R and not in GA-S or NC-S *A. palmeri* individuals. PCR on gDNA of 3 GA-R and 3 GA-S *A. palmeri* with primers A) 5′ MITE.F by 5′ MITE.R, B) 3′ MITE.F by 3′ MITE.R, C) 5′ MITE.F by Ex1R and D) Ex8F by 3′ MITE.R; PCR on gDNA of 16 GA-S and 1 GA-R *A. palmeri* with primers E) 5′ MITE.F by Ex1R, F) Ex8F by 3′ MITE.R, and G) Ex1F by Ex1R as a positive PCR control; H) PCR on gDNA of 5 NC-S (North Carolina) and 1 GA-R *A. palmeri* with primers (left to right) Ex1F by Ex1R as a positive PCR control, 5′ MITE.F by Ex1R, and Ex8F by 3′ MITE.R. Negative controls (templates without primers and primers without template) were evaluated separately and no PCR products were observed.
(TIF)

Figure S6 Schematic diagram of steps used in *EPSPS* intron analysis and genomic sequencing of *A. palmeri* populations.
(DOCX)

Table S1 PCR primers used in experiments to sequence introns, conduct qPCR on introns, synthesize Southern blot probes, and amplify MITE-homologous sequences.
(DOCX)

Table S2 Contigs from 454 sequencing that align with fosmid reference sequence (see Figure 3) have a high number of hits. Raw read hits for each contig were normalized for size to 1000 bp to facilitate comparisons across contigs.
(DOCX)

Methods S1. (DOCX)
