# Supplementary material for: Identification of Genetic Elements Associated with EPSPS Gene Amplification
Source: PLoS One. 2013 Jun 10;8(6):e65819. doi: 10.1371/journal.pone.0065819 (PMC3677901; doi:10.1371/journal.pone.0065819)
Supplement: Figure S1 — Sequence alignment of EPSPS genomic sequence (exon 4 to exon 6) from representative glyphosate-resistant (R) and –susceptible (S) A. palmeri cloned PCR products. Primers Ex4F and Ex6R1 are underlined, and XhoI polymorphism is highlighted with a square. Intron sequences are in lower case. (DOCX) [file pone.0065819.s001.docx]

Figure S1. Sequence alignment of *EPSPS* genomic sequence (exon 4 to exon 6) from representative glyphosate-resistant (R) and –susceptible (S) *A. palmeri* cloned PCR products. Primers Ex4F and Ex6R1 are underlined, and *Xho*I polymorphism is highlighted with a square. Intron sequences are in lower case.

R1.a GTGATAGTTGGGACAGGTTCTACATTCGAGGTGGTCAGAAATACAAgtaagtctctcatc 60

S3.a GTGATAGTTGGGACAGGTTCTACATTCGAGGTGGTCAGAAATACAAgtaagtctctcatc 60

S1.a GTGATAGTTGGGACAGGTTCTACATCCGAGGTGGTCAGAAATACAAgtaagtttctcatc 60

************************* ************************** *******

R1.a ttatattacatgtccttttaacgtgtctccattagtagactgaaaacacatgtaaataca 120

S3.a ttatattacatgtccttttaacgtgtctccattagtagactgaaaacacatgtaaataca 120

S1.a ttattttacatgtccttttaacgtgtctccattagtagactgaaaacacatgtaaatgca 120

****:****************************************************.**

R1.a tcagATCTCCTGGAAAGGCATATGTTGAGGGTGATGCTTCAAGTGCTAGCTACTTCCTAG 180

S3.a tcagATCTCCTGGAAAGGCATATGTTGAGGGTGATGCTTCAAGTGCTAGCTACTTCCTAG 180

S1.a tcagATCTCCTGGAAAGGCATATGTAGAGGGGGACGCTTCTAGTGCTAGCTACTTCCTAG 180

*************************:***** ** *****:*******************

R1.a CCGGAGCCGCCGTCACTGGTGGGACTGTCACTGTCAAGGGTTGTGGAACAAGCAGTTTAC 240

S3.a CCGGAGCCGCCGTCACTGGTGGGACTGTCACTGTCAAGGGTTGTGGAACAAGCAGTTTAC 240

S1.a CAGGAGCCGCCGTCACTGGTGGGACTGTGACTGTCAAGGGTTGTGGAACAAGCAGTTTAC 240

*.************************** *******************************

R1.a AGgtataatgttaacccttacccttcacattgttctgctaaattctagaggaccctttca 300

S3.a AGgtataatgttaacccttacccttcacattgttctgctaaattctagaggaccctttca 300

S1.a AGgtataatgttaacccttacccttcacattgttctgctaaattctagagaaccctttca 300

**************************************************.*********

R1.a attctgggtgggataagcacggcaatttgaccgcaaaaaaattgcaaaattattctgctg 360

S3.a attctgggtgggataagcacggcaatttgaccgcaaaaaaattgcaaaattattctgctg 360

S1.a attctgggtgggataagcacgacaatttgactgc-aaaaaattgcaaaattattctgctg 359

*********************.********* ** *************************

*Xho* I

R1.a atagaacatctcgagatgagatcatattgagttttggcgtcaacataaacctaatcaaat 420

S3.a atagaacatctcgagatgagatcatattgagttttggcgtcaacataaacctaatcaaat 420

S1.a atagaacatatcgagatgagatcatattgagttttggcgtcaacataaacctaatcaaat 419

*********.**************************************************

R1.a aatgaaaaataca---aacatcatatggtttcttttgtctttatgactagacactctcta 477

S3.a aatgaaaaataca---aacatcatatggtttcttttgtctttatgactagacactctcta 477

S1.a aatgaaaaatacaaacaacatcatatggtttcttttgtctttatgactagacactctcta 479

************* ********************************************

R1.a ttattccttgattgggatcttatttgaaattgctgtgtagcctacacctcatgttcagat 537

S3.a ttattccttgattgggatcttatttgaaattgctgtgtagcctacacctcatgttcagat 537

S1.a ttattccttgattgggatcttatttgaaattgttgtgtagcctacacctcatgttcagat 539

******************************** ***************************

R1.a tttgttcgtataccagacttttcttgattgggatcttatttgtcccctggattttgcata 597

S3.a tttgttcgtataccagacttttcttgattgggatcttatttgtcccctggattttgcata 597

S1.a tttgttcgtataccagacttttcttgattgggatcttatttgtcccctggattttgcata 599

************************************************************

R1.a gGGTGATGTAAAATTTGCCGAAGTTCTTGAGAAGATGGGTTGCAAGGTCACCTGGACAGA 657

S3.a gGGTGATGTAAAATTTGCCGAAGTTCTTGAGAAGATGGGTTGCAAGGTCACCTGGACAGA 657

S1.a gGGTGATGTAAAATTTGCCGAAGTTCTTGAGAAGATGGGTTGCAAGGTCACCTGGACAGA 659

************************************************************

R1.a GAATAGTGTAACTGTTACTGGACCACCCAGGGATTCATCTGGAAAGAAACATCTGCGTGC 717

S3.a GAATAGTGTAACTGTTACTGGACCACCCAGGGATTCATCTGGAAAGAAACATCTGCGTGC 717

S1.a GAATAGTGTAACTGTTACTGGACCGCCCAGGGATTCATCTGGAAAGAAACATCTGCGTGC 719

************************.***********************************

R1.a TATCGACGTCAACATGAACAAAATGCCAGATGTTGCTATGACTCTTGC 765

S3.a TATCGACGTCAACATGAACAAAATGCCAGATGTTGCTATGACTCTTGC 765

S1.a TATCGACGTCAACATGAACAAAATGCCAGATGTTGCTATGACTCTTGC 767

************************************************
