# Supplementary material for: Identification of Genetic Elements Associated with EPSPS Gene Amplification
Source: PLoS One. 2013 Jun 10;8(6):e65819. doi: 10.1371/journal.pone.0065819 (PMC3677901; doi:10.1371/journal.pone.0065819)

Figure S2. A) Alignment of fosmid consensus sequence (MS-R), 454 consensus sequence (GA-R), and the probes used for Southern blots (5’ MITE and 3’ MITE from GA-R), showing a sequence divergence point between MS-R and GA-R upstream of *EPSPS*. A 13 bp imperfect Terminal Inverted Repeat (TIR) is underlined with one non-matching base in lower case, and a 3 bp duplicated sequence similar to known Target Site Duplications (TSD) is shown in bold and italics. A TATA box in the fosmid sequence is located 496 bp from the *EPSPS* start codon. B) PCR confirmation of 454 contig 00009 (GA-R) assembly upstream of *EPSPS* and divergence from the fosmid sequence (MS-R). Primers MSFor1 and MSFor2 did not produce PCR amplicons with primer 1R1 on gDNA from 3 GA-R individuals, but primers GAFor1 and GAFor2 produced amplicons of the expected sizes (823 and 628 bp) with primer 1R1.

**A**

5’ alignment:

>----Primer 5’ MITE.F------>

5' MITE probe (1) --------------------------------------tGAAAGATCATACTCTAACCTGCAATAGTAGGTCAAATTTCA

EPSPS 454 Consensus (455) TTTTGGGTTGAATGTTTATTTTATGGAAT***TAA***AGAATAtGAAAGATCATACTCTAACCTGCAATAGTAGGTCAAATTTCA

EPSPS Fosmid Consensus (738) CAGTAGCTGG---GTTTATAAAGAGGCATTGAAGAA---GACATTGAAGAAGATAACCTACTAAAATAGGTCAAATTTCA

*** ** * * * ****** * * * **************

TATA Box

<--Primer-

5' MITE probe (43) TTTGGGGGTGCCACGAGCAAATACACTTGAAAGGTGAGATTATTCATAAATAATCAATACTTGGGATTATTCACATAGGT

EPSPS 454 Consensus (535) TTTGGGGGTGCCACGAGCAAATACACTTGAAAGGTGAGATTATTCATAAATAATCAATACTTGGGATTATTCACATAGGT

EPSPS Fosmid Consensus (812) TTTGGGGGTGCCACGAGCAAATACACTTGAAAGGTGGGATTATTCATAAATAATCAATACTTGGGATTATTCACATAGGT

************************************ *******************************************

----5’ MITE.R---<

5' MITE probe (123) TTGCGAATAGTTCGGAT---------

EPSPS 454 Consensus (615) TTGCGAATAGTTCGGATTATTCCCAA

EPSPS Fosmid Consensus (892) TTGCGAATAGTTCGGATTATTCCCAA

*****************

3’ alignment:

>----Primer 3’ MITE.F---->

3' MITE probe (1) TGGAAAGTTTTCATCCCAGTTCCCACATTTACTCCTTAAAACCCCACTTTCCTTACTTTACACTACTATTTAATTATTTT

EPSPS 454 Consensus (11623) TGGAAAGTTTTCATCCCAGTTCCCACATTTACTCCTTAAAACCCCACTTTCCTTACTTTACACTACTATTTAATTATTTT

EPSPS Fosmid Consensus (11901) TGGAAAGTTTTCATCCCAGTTCCCACATTTACTCCTTAAAACCCCACTTTCCTTACTTTACACTACTATTTAATTATTTT

********************************************************************************

3' MITE probe (81) CTCTCTTATACTTCCAATACAAGTATTACATTATACTATTATTTAATTATTTTTTCTCTCATACTTTCAATACAATCATT

EPSPS 454 Consensus (11703) CTCTCTTATACTTCCAATACAAGTATTACATTATACTATTATTTAATTATTTTTTCTCTCATACTTTCAATACAATCATT

EPSPS Fosmid Consensus (11981) CTCTCTTATACTTCCAATACAAGTATTACATTATACTATTATTTAATTATTTTTTCTCTCATACTTTCAATACAATCATT

********************************************************************************

3' MITE probe (161) ACTTTTCACTACTATGAAGTAATTAAAATAATACCCATTACCACCAAAGATTCCATTTTTCTTAATCTTGGTGAAAAACC

EPSPS 454 Consensus (11783) ACTTTTCACTACTATGAAGTAATTAAAATAATACCCATTACCACCAAAGATTCCATTTTTCTTAATCTTGGTGAAAAACC

EPSPS Fosmid Consensus (12061) ACTTTTCACTACTATGAAGTAATTAAAATAATACCCATTACCACCAAAGATTCCATTTTTCTTAATCTTGGTGAAAAACC

********************************************************************************

<------Primer 3’ MITE.R---------<

3' MITE probe (241) CAAATAGGAACATCAAAAAGGAACGGAGGGAGTATCTTTCtTATTC***TAA***CCAAATG----------------------

EPSPS 454 Consensus (11863) CAAATAGGAACATCAAAAAGGAACGGAGGGAGTATCTTTCtTATTC***TAA***CCAAATGTAATACTCTTTCCAACTCTCTT

EPSPS Fosmid Consensus (12141) CAAATAGGAACATCAAAAAGGAACGGAGGGAGTATCTTTCtTATTC***TAA***CCAAATGTAATACTCTTTCCAACTCTCTT

*******************************************************************************

**B**


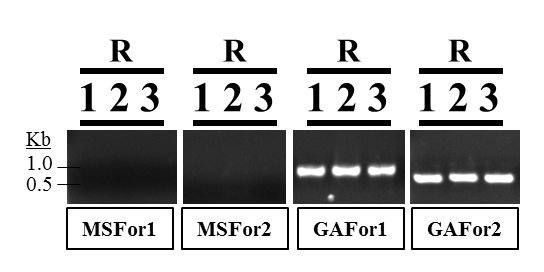

Supplement: Figure S2 — Alignment of fosmid consensus sequence (MS-R), 454 consensus sequence (GA-R), and the probes used for Southern blots (5′ MITE and 3′ MITE from GA-R). A 13 bp imperfect Terminal Inverted Repeat (TIR) is underlined with one non-matching base in lower case, and a 3 bp duplicated sequence similar to known Target Site Duplications (TSD) is shown in bold and italics. (DOCX) [file pone.0065819.s002.docx]
