# Supplementary material for: Identification of Genetic Elements Associated with EPSPS Gene Amplification
Source: PLoS One. 2013 Jun 10;8(6):e65819. doi: 10.1371/journal.pone.0065819 (PMC3677901; doi:10.1371/journal.pone.0065819)
Supplement: Figure S3 — A 256 bp imperfect inverted repeat identified in fosmid sequence from MS-R gDNA; A, alignment showing identity between inverted repeat on ends of the putative transposon (Figure 4) and B, sequence of the inverted repeat. (DOCX) [file pone.0065819.s003.docx]

Figure S3. A 256 bp imperfect inverted repeat identified in fosmid sequence from MS-R gDNA; A, alignment showing identity between inverted repeat on ends of the putative transposon (Figure 4) and B, sequence of the inverted repeat.


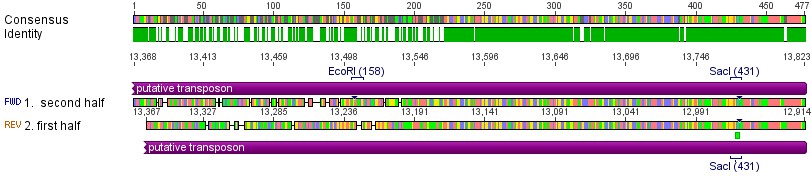
**A**

**B**

1 aacttttttttttaaaacttttttttaaaaatatttggctttttaagagctccctcaaattatttgcaaa

||||||||||||| |||||||||||||||||||||||||||||||||||||||||||||||||||||||

910 ttgaaaaaaaaaaaattgaaaaaaaatttttataaaccgaaaaattctcgagggagtttaataaacgttt

71 aataagcttctacgcataatgggctgggggctgggttttgagctggtaatgggccgctgttagtacaagc 271

||||||||||||||| ||| ||||||||||||||||||||||||||||||||||||||||||||||||||

840 ttattcgaagatgcgcattgcccgacccccgacccaaaactcgaccattacccggcgacaatcatgttcg 640

141 ggacaattcattatgggctgatttaccaatcggccgctgtttatatcagcgggccattggtcttcatgga

|| ||||||||| |||| |||| |||||||||||||||||||||||||||||||||||||||||||||

770 cccgttaagtaaaaccccgataaaaggttagccggcgacaaatatagtcgcccggtaaccagaagtacct

211 atgggccgcattcggccgctgtttgtatcagcgggccattgggctt 256

|||||||||||||||||||||||| |||||||||||||||||||||

700 tacccggcgtaagccggcgacaaatatagtcgcccggtaacccgaa 655
