# Supplementary figures and images for: Identification of Genetic Elements Associated with EPSPS Gene Amplification
Source: PLoS One. 2013 Jun 10;8(6):e65819. doi: 10.1371/journal.pone.0065819 (PMC3677901; doi:10.1371/journal.pone.0065819)

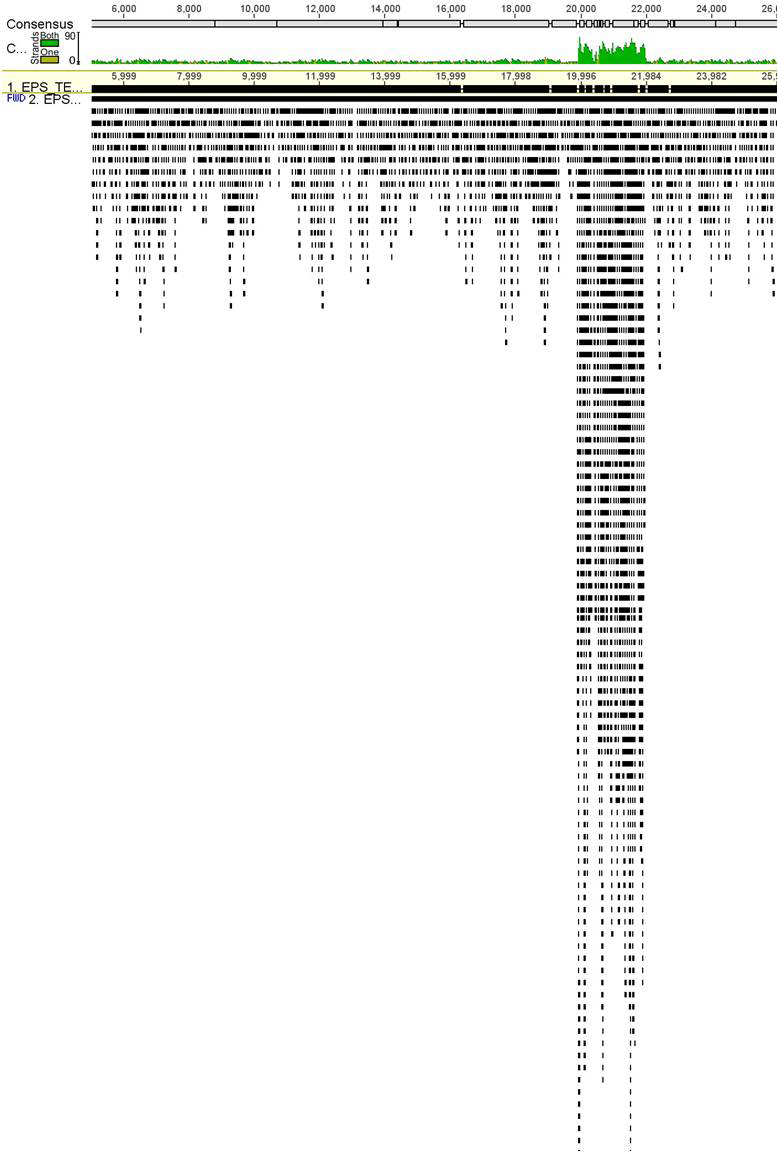

Supplement: Figure S4 — Assembly of Illumina reads to the fosmid reference sequence, and the presence of read stacking in a region from approximately 20,000 bp until 22,000 bp. The sequence in this region contains a repeated 551 bp sequence. (TIF) [file pone.0065819.s004.tif]

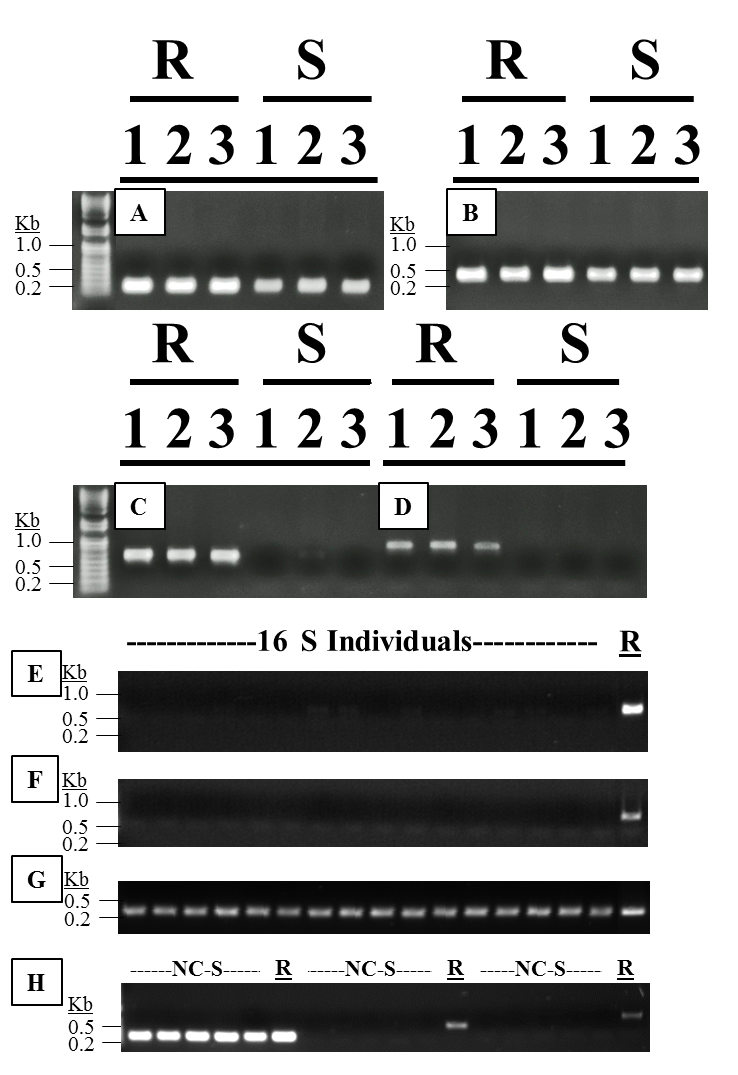

Supplement: Figure S5 — PCR evidence for existence of putative MITE sequences in both GA-R and GA-S, but present flanking EPSPS only in GA-R and not in GA-S or NC-S A. palmeri individuals. PCR on gDNA of 3 GA-R and 3 GA-S A. palmeri with primers A) 5′ MITE.F by 5′ MITE.R, B) 3′ MITE.F by 3′ MITE.R, C) 5′ MITE.F by Ex1R and D) Ex8F by 3′ MITE.R; PCR on gDNA of 16 GA-S and 1 GA-R A. palmeri with primers E) 5′ MITE.F by Ex1R, F) Ex8F by 3′ MITE.R, and G) Ex1F by Ex1R as a positive PCR control; H) PCR on gDNA of 5 NC-S (North Carolina) and 1 GA-R A. palmeri with primers (left to right) Ex1F by Ex1R as a positive PCR control, 5′ MITE.F by Ex1R, and Ex8F by 3′ MITE.R. Negative controls (templates without primers and primers without template) were evaluated separately and no PCR products were observed. (TIF) [file pone.0065819.s005.tif]
