# Supplementary material for: Identification of Genetic Elements Associated with EPSPS Gene Amplification
Source: PLoS One. 2013 Jun 10;8(6):e65819. doi: 10.1371/journal.pone.0065819 (PMC3677901; doi:10.1371/journal.pone.0065819)
Supplement: Figure S6 — Schematic diagram of steps used in EPSPS intron analysis and genomic sequencing of A. palmeri populations. (DOCX) [file pone.0065819.s006.docx]

GA-R Population MS-R Population

↓ **↓** **↓**

Sequencing and qPCR using qPCR for gDNA *EPSPS* qPCR for gDNA *EPSPS*

Intron-specific primers copy number copy number

**↓** **↓**

gDNA 454 Sequencing and Fosmid library, Illumina Sequencing and

Assembly (1 individual) Assembly (1 individual)

**↓** **↓**

Blots: *EPSPS* and MITE probes **←** Sequence Analysis and Identification of Genetic Elements

(GA-R & GA-S only)

↓

Sequence Alignments and Comparison
