# Supplementary material for: Identification of Genetic Elements Associated with EPSPS Gene Amplification
Source: PLoS One. 2013 Jun 10;8(6):e65819. doi: 10.1371/journal.pone.0065819 (PMC3677901; doi:10.1371/journal.pone.0065819)
Supplement: Table S1 — PCR primers used in experiments to sequence introns, conduct qPCR on introns, synthesize Southern blot probes, and amplify MITE-homologous sequences. (DOCX) [file pone.0065819.s007.docx]

Table S1. PCR primers used in experiments to sequence introns, conduct qPCR on introns, synthesize Southern blot probes, and amplify MITE-homologous sequences.

| Primer Name | 5’ to 3’ sequence | Location |
| --- | --- | --- |
| Ex4F | GTGATAGTTGGGACAGGTTCTACA | Exon 4 |
| Ex6R1 | GCCAGATGTTGCTATGACTCTTGC | Exon 6 |
| In5F | TGTAGCCTACACCTCATGTTCAGAT | Intron 5 |
| Ex6R2 | TACACTATTCTCTGTCCAGGTGACCTT | Exon 6 |
| 5’ MITE.F | TGAAAGATCATACTCTAACCTGCAATAGTAG | 5’ flanking MITE |
| 5’ MITE.R | ATCCGAACTATTCGCAAACCTATGTGA | 5’ flanking MITE |
| 3’ MITE.F | TGGAAAGTTTTCATCCCAGTTCCCAC | 3’ flanking MITE |
| 3’ MITE.R | CATTTGGTTAGAATAAGAAAGATACTCCCTCCG | 3’ flanking MITE |
| Ex1r | GGACAGATGAAGGTTTCTCAGCTGCAGC | Exon 1 |
| Ex1f | GGCTCAAGCTACTACCATCAACAATGG | Exon 1 |
| Ex8r | CAAAGTAGTCCGGGAAGGTTTTACGGGTG | Exon 8 |
| Ex8f | GATCTGATTACTGTGTGATCACTCCGCC | Exon 8 |
| MSFor1 | atcctgattgcatttcatggtttgat | Upstream of 5’ MITE in MS-R fosmid sequence |
| MSFor2 | atgatttggtggtcactagtcttggtgt | Upstream of 5’ MITE in MS-R fosmid sequence |
| GAFor1 | ttcgcacagagcagcaataccac | Upstream of 5’ MITE in GA-R 454 sequence |
| GAFor2 | gaacaggaaatgaagttaagggtatgcct | Upstream of 5’ MITE in GA-R 454 sequence |
| AW21 | CAGGTTCTACATTCGAGGTG | Exon 4 |
| AW22 | GTGACAGTCCCACCAGTG | Exon 5 |
