# Supplementary material for: Identification of Genetic Elements Associated with EPSPS Gene Amplification
Source: PLoS One. 2013 Jun 10;8(6):e65819. doi: 10.1371/journal.pone.0065819 (PMC3677901; doi:10.1371/journal.pone.0065819)
Supplement: Table S2 — Contigs from 454 sequencing that align with fosmid reference sequence (see Figure 3) have a high number of hits. Raw read hits for each contig were normalized for size to 1000 bp to facilitate comparisons across contigs. (DOCX) [file pone.0065819.s008.docx]

Table S2. Contigs from 454 sequencing that align with fosmid reference sequence (see Figure 3) have a high number of hits. Raw read hits for each contig were normalized for size to 1000 bp to facilitate comparisons across contigs.

| Contig | Size (bp) | Normalized Hits to Contig |
| --- | --- | --- |
| 00009 | 14268 | 230 |
| 00023 | 5512 | 428 |
| 00026 | 5236 | 483 |
| 00042 | 4274 | 445 |
| 08451 | 848 | 159 |
| 09666 | 787 | 6235 |
| 18781 | 410 | 966 |
| 21393 | 335 | 36 |
| 27320 | 204 | 201 |
| 27936 | 191 | 1812 |
| 28356 | 183 | 12530 |
| 32277 | 117 | 6650 |
| 33258 | 102 | 7696 |
