# Supplementary material for: Identification of Genetic Elements Associated with EPSPS Gene Amplification
Source: PLoS One. 2013 Jun 10;8(6):e65819. doi: 10.1371/journal.pone.0065819 (PMC3677901; doi:10.1371/journal.pone.0065819)
Supplement: Methods S1. — (DOCX) [file pone.0065819.s009.docx]

Supporting Information – Materials and Methods

***Fosmid Library and Sequencing***

Genomic DNA was extracted using the Masterpure^TM^ DNA purification kit (Epicentre) and the fosmid library was constructed using the CopyControl^TM^ Fosmid Library Production Kit with pCC1FOS^TM^ Vector (Epicentre). Random-sheared and end-repaired DNA inserts were ligated into the fosmid vector according to kit instruction. All clones were glycerol stocked in 200 µL Wu broth (6.27 g/L K_2_HPO_4_, 1.8 g/L KH_2_PO_4_, 0.5 g/L NaCitrate, 0.9 g/L (NH_4_)_2_SO_4_, 10 g/L tryptone, 5 g/L yeast extract, 10 g/L NaCl, 44 mL/L glycerol). Fosmids were screened by pooled PCR using primers amplifying from exon 4 to exon 5 of *EPSPS* (primers AW21 and AW22, Table S1). After glycerol stocking individual colonies, plates were flushed with 1 mL LB (1% w/v tryptone, 0.5% w/v yeast extract, and 0.5% w/v NaCl) with 12.5 µg/mL chloramphenicol and 2 µL/mL autoinduction solution (Epicentre) to pool the colonies. Pools were incubated overnight at 37°C. PCR reactions were performed using the LA PCR kit version 2.1 (Fisher). Reactions were performed in a 25 µL volume containing 5 µL template, 400 nM each primer (AW21 and AW22), 400 µM dNTPs, 2.5 mM Mg^2+^, 1 x buffer, and 1.25 units of polymerase. Cycle conditions consisted of 94°C for 3 min; 30 cycles of 94°C for 30 s, 55°C for 30s, and 72°C for 1 min; and final extension of 72°C for 5 min. PCR products were analyzed on a 1% agarose gel in 1 X TAE (0.04 M Tris-acetate, 0.001 M EDTA). For plates with positive colonies, smaller pools were generated and screened again by PCR. For positive smaller pools, individual colonies were screened.

The protocol for isolation of fosmid DNA was provided by the USDA-ARS Genomics and Bioinformatics Research Unit (GBRU), Stoneville, MS, USA. To prepare fosmids for Sanger sequencing and Illumina library construction, the fosmids were gel purified using the manufacturer’s protocol (Epicentre). The quantity and quality of the gel purified DNA was examined by gel electrophoresis.

***Southern Blot Analysis***

Genomic DNA from one GA-R and one GA-S individual was extracted using the CTAB method as previously described [[1](#_ENREF_1)] with an initial phenol:chloroform:isoamyl alcohol (25:24:1) separation, followed by 2 subsequent chloroform:isoamyl alcohol (24:1) separations and an RNAse digestion. Overnight restriction digests of 11 μg (R) and 2×10 μg (S) gDNA were performed at 37° C in 200 μL volume using *Eco*R I, *Xho* I, *Bam*H I, and *Sac* I in the manufacturer recommended buffers (New England Biolabs). Digested gDNA aliquots were separated using chloroform:isoamyl alcohol (24:1) and precipitated using 100% ethanol and 0.5M sodium acetate. Digested gDNA samples were gel-electrophoresed in 0.7% agarose at 25V for 19 h. The S gDNA digests were loaded as 20 μg per lane, and the R gDNA digests were loaded in one lane with 1 μg and in a second lane with 10 μg gDNA. The gel was treated with initial depurination in 0.25M HCl for 30 min, followed by denaturation in 0.5M NaOH/1.5 M NaCl (2×15 min), followed by neutralization in 1.5M NaCl/0.5 M Tris-Cl, pH 7.0 (2×20 min) [[2](#_ENREF_2)]. Transfer to a positively charged nylon membrane was conducted overnight using the saline transfer method in 20X SSC (3.0 M NaCl and 0.3 M sodium citrate, pH 7.0). The membrane was air-dried and the DNA was immobilized to the membrane using 40 sec exposure to UV light.

Probes were PCR amplified using a high fidelity DNA polymerase (Qiagen HotStar High Fidelity) in 50 μL reactions with 1X buffer, 200 μM each dNTP, 0.2 μM each primer, 0.5 Units polymerase, and 10 ng GA-R gDNA, with initial denaturation at 95° C for 5 min and then 30 cycles with 94° C for 15 sec, 55° C for 1 min, and 72° C for 1 min, and a final extension at 72° C for 10 min. Probes were amplified for Exon 1 and Exon 8 of *EPSPS*, and for two DNA sequences identified from regions flanking *EPSPS*, one in the 5’ direction and one in the 3’ direction from *EPSPS* (primers in Table S1). PCR products were analyzed on 1% agarose gel and gel-purified for cloning using the TOPO TA PCR cloning kit (Invitrogen). One positive clone was selected for each probe, plasmids were purified from overnight LB cultures (Qiagen Plasmid Mini Kit) and the insert was digested from the plasmid using *Eco*R I (New England BioLabs). Probes were labeled using ^33^P-dCTP (Hartmann Analytic, Braunschweig, Germany).

Membrane pre-hybridization was conducted at 42° C in hybridization buffer (DIG Easy Hyb, Roche). Labeled probe was added to pre-warmed hybridization buffer and hybridization was carried out overnight at 42° C. All hybridization steps were carried out in sealed hybridization bags (Roche). The membrane was washed (2×5 min) in low stringency wash (2X SSC, 0.1% SDS) at room temperature and washed (2×15 min) in high stringency wash (0.5X SSC, 0.1% SDS) at 65° C. The membrane was sealed in plastic wrap and exposed for 18 h to an imaging plate (BAS-IP MS 2040, Fuji Film), and the image was scanned from the imaging plate using a phosphorimager (Fujix BAS 1000). The membrane was then stripped using 2×15 min washes in stripping buffer (0.25 NaOH and 0.1% SDS), rinsed in 2X SSC, and evaluated for satisfactory removal of labeled probe using exposure to an imaging plate for 4 h.

REFERENCES

1. Gaines TA, Zhang W, Wang D, Bukun B, Chisholm ST, et al. (2010) Gene amplification confers glyphosate resistance in *Amaranthus palmeri*. Proceedings of the National Academy of Sciences of the United States of America 107: 1029-1034.

2. Ausubel FM, Brent R, Kingston RE, Moore DD, Seidman JG, et al., editors (1993) Current Protocols in Molecular Biology: John Wiley & Sons.
